# Cultural awareness and competence of pharmacy educators and learners from the perspective of pharmacy students at Qatar University: A mixed-methods approach

**DOI:** 10.1371/journal.pone.0243095

**Published:** 2020-12-02

**Authors:** Banan Mukhalalati, Ma’al Shahrour, Sara Rabie, Ahmed Awaisu, Sara Elshami, Feras Alali

**Affiliations:** Health Cluster, College of Pharmacy, Qatar University, Doha, Qatar; Lingnan University, HONG KONG

## Abstract

**Background:**

Since healthcare professional educators and practitioners in Qatar are culturally diverse, the impact of this diversity on the education and training of healthcare students should be evaluated. This study, therefore, aims at examining pharmacy students’ perspectives on the level of cultural awareness and competence of pharmacy educators and learners at Qatar University and the influence of cultural diversity on pharmacy education in Qatar.

**Methods:**

A convergent mixed-methods design was adopted. The Cultural Awareness Scale (CAS) was utilized in the quantitative phase, which was administered on 122 pharmacy students at Qatar University College of Pharmacy (QU CPH), of whom 70 responded. The qualitative phase comprised four focus groups with a total of 23 students. The quantitative and qualitative data were collected concurrently, and the results were integrated.

**Results:**

The findings suggest that the QU CPH is an institution of a culturally diverse community. Educators and students alike are generally culturally aware and sensitive; however, demonstration of a holistic awareness was hindered by a few barriers. This study suggests curricular changes to reinforce cultural competence, cultural inclusiveness, and the preservation of Qatar’s cultural identity and values in the educational environment.

**Conclusions:**

The internationalization of pharmacy education in Qatar has inspired students and educators alike to achieve new dimensions of cultural awareness. To infuse passion and enthusiasm in learning while maintaining Qatar’s cultural values and identity, healthcare professional educators, researchers, and policymakers are required to collaborate to promote culturally sensitive pharmacy education.

## Introduction

In healthcare professional education, it is essential to incorporate cultural dimensions into the processes of teaching and learning to assist students in the application of acquired knowledge to their own cultural milieu and answer their queries, a process which could otherwise be hindered by cultural diversity [1]. In addition, health profession schools ought to prepare students to be culturally competent healthcare practitioners who are able to equitably meet the demands of a population with diverse values, beliefs, and customs, hence improving health outcomes and eliminating disparities [2–5]. Tylor (1871) defined culture as a “complex whole which includes knowledge, beliefs, arts, morals, law, customs, and any other capabilities and habits acquired by man as a member of society” [6]. As described, culture encompasses a large and complex aspect of human life, and language is certainly crucial for communication and thus is an important element of culture [7]. As language awareness is obviously essential for successful communication in the delivery of health care, health professional education programs ought to ensure that students are equipped to provide adequate linguistic care to patients [8]. Furthermore, according to O'Connell (2013), cultural diversity should encompass not only race and ethnicity, but also socioeconomic status, rural and urban background, age, gender, sexual orientation, ability, and other life experiences [9]. Thus, diversity among members of a society in these aspects is counted as cultural diversity.

Literature has shown that it is very challenging for healthcare professional educators to incorporate cultural dimensions in an academic environment that is highly diverse, as individual students have different needs and distinctive interpretations of the knowledge imparted to them [10–12]. Furthermore, cultural diversity can compromise the effectiveness of learner-instructor communication. Since communication is a core element of effective teaching and learning processes, poor communication can negatively impact students’ learning outcomes. On the other hand, cultural diversity can prove beneficial in engaging a country’s workforce with that of the world and preparing healthcare students to be more culturally competent and aware [13]. Healthcare providers who are exposed to culturally diverse and inclusive educational environments are more likely to demonstrate a strong inclination toward healthcare equity and positive interracial interactions compared with those who were minimally exposed or not exposed to the same [13, 14].

To overcome the daily challenges posed by diversity, new concepts have been developed for healthcare providers and educators. The first is “cultural humility” that entails the healthcare providers to self-reflect their own cultural identity and beliefs before learning about others’ cultures [15, 16]. The second is “cultural competence” which is defined as “the attitudes, knowledge, and skills necessary for providing quality care to diverse populations” [17]. The third is “cultural pluralism” which entails “a mixing of different cultures in which each culture retains its own unique identity” [18]. These concepts are necessary for assisting educators in today’s globalized educational atmosphere to constantly learn from other cultures and to optimize their own knowledge, skills, and attitudes. This will eventually help educators tailor their educational strategies according to the diverse needs of students and therefore enable them to have access to equitable learning experiences in such diverse societies [19–21].

Cultural diversity is a prominent and evolving phenomenon in Qatar, an Islamic Arab State, and one of the Gulf Coast Cooperation (GCC) member countries, whose laws and customs follow Islamic and Arabic traditions [22]. The large majority of Qatar’s population comprises expatriates, at 88.4%, while the people of Arab descent constitute only a small minority [23, 24]. The majority of expatriates come from India (22%), Bangladesh (12.5%), and Nepal (12.5%) [25, 26]. Although Arabic is the official language of Qatar, there are around 190 different languages spoken [23, 24] with English being the most widely used, especially in schools and businesses [25]. In addition to Muslims, there is a significant presence of Christians, Hindus, Buddhists, and Baha’is in the peninsular country [25]. This makes Qatar one of the most multicultural societies in the world. Furthermore, in recent years, Qatar has witnessed the internationalization of its healthcare delivery system and healthcare professional education with significant investments and reforms in the health and education sectors based on Western models. As a result, employment opportunities in the education and health sectors in Qatar have attracted professionals from diverse cultures around the world. However, according to the Qatar National Vision (QNV) 2030, it is important to minimize the conflict between traditional cultural values and identities and the modern patterns of social life nurtured by cultural diversity [27]. As such, Qatari society ought to observe and preserve the fundamental principles of Islam and the Arab identity, respectively.

A systematic review of medical education in Qatar revealed clear trends of globalization [28]. For instance, in 2009, Weil Cornell Medical College in Qatar established the Center for Cultural Competence in Health Care to address issues pertaining to cross-culture in healthcare education and practice besides training healthcare providers and educators in dealing with cultural diversity and achieving cultural competency. Such skills can be acquired by considering the perceptions of various stakeholders, including education stakeholders, about cultural variation. Therefore, incorporating cultural awareness issues in the curricula of health professional training programs in Qatar, including pharmacy programs, is important [24].

Diversity is one of the core values of Qatar University (QU), as it promotes a culture that values multireligious and multicultural principles, and considers diversity among students and faculty to be a pillar of strength that enriches its educational and work environment [29]. Notably, QU was ranked number one in the International Outlook indicator in the 2019 overall Times Higher Education World University Rankings list, and has received this honor for the fourth time in a row [30]. In QU, there are approximately 22,461 students with Qatari registrants being 2 times higher than non-Qataris (mostly from Arab (non-GCC countries), Asian, and GCC countries), and females being 3.5 times higher than males [29]. On the other hand, there were around 1000 faculty members, mainly from Arab (non-GCC) countries, GCC countries, North America, Europe, and Asia. The university hosts the health cluster, which includes four colleges: College of Pharmacy, College of Medicine, College of Health Sciences, and College of Dental Medicine. The College of Pharmacy (CPH), established in 2006, is the first and only of its kind to offer a pharmacy degree program in the State of Qatar. In 2009, the CPH became the first international pharmacy program to receive accreditation by the Canadian Council for Accreditation of Pharmacy Programs (CCAPP). The CPH offers a 5-year BSc (Pharmacy) degree program, a 6-year Doctor of Pharmacy (Pharm-D) degree program, a 2-year MSc (Pharm) degree program, and a 4-year Doctor of Philosophy (PhD) program in pharmaceutical sciences. The college’s vision is to promote healthcare in Qatar, the Middle East, and around the globe through quality and innovative pharmacy education, research, and service. Since its establishment and until the spring of 2019, the CPH accepted only female students in the undergraduate program with an average class size of 22 to ensure excellent faculty-to-student ratio [31]. This makes acceptance to a pharmacy degree program very competitive, it being based on academic performance and the demonstration of motivation to be a competent healthcare professional. Applicants to the pharmacy programs must demonstrate fluency in English, measured by a minimum TOEFL score of 500 (or IELTS 5.5), as instruction and assessment are in English. The majority of students (89%) who were enrolled in the BSc program were native Arabic speakers from Qatar (constituting 17% of total admissions) and other Arab countries such as Egypt, Jordan, Sudan, and Syria. Non-Arab residents from Iran, Somalia, Canada, USA, Germany, India, Eritrea, Philippines, Mali, Djibouti, and Bangladesh contributed to the diversity of the CPH. On the other hand, the 2017–2020 college’s records indicate that the CPH faculty members comprised both men and women, with more males (25) than females (18). The majority of the CPH faculty are from non-Arab Western countries such as Canada, the UK, and the USA, in addition to African countries such as Ghana and Nigeria. Arab nationals constituted only one-third of the faculty. The CPH’s instructor and student nationalities are presented in Table 1.

**Table 1:** The CPH’s instructor and student nationalities.

Nationality	Faculty N (%)	Students N (%)
**Arabs**	**15 (36%)**	**290 (89%)**
Qatari	3 (7%)	55 (17%)
Saudi	0 (0%)	2 (0.6%)
Bahraini	0 (0%)	2 (0.6%)
Yemeni	0 (0%)	7 (2.1%)
Iraqi	0 (0%)	11 (3.4%)
Lebanese	4 (10%)	1 (0.3%)
Syrian	2 (5%)	30 (9.2%)
Jordanian	2 (5%)	30 (9.2%)
Palestinian	0 (0%)	22 (6.7%)
Sudanese	1 (2%)	39 (12%)
Egyptian	2 (5%)	71 (22%)
Algerian	0 (0%)	11 (3.4%)
Libyan	0 (0%)	3 (0.9%)
Tunisian	0 (0%)	4 (1.2%)
Moroccan	0 (0%)	2 (0.6%)
**Non-Arabs**	**27 (64%)**	**35 (11%)**
American	3 (7%)	3 (0.9%)
British	6 (14%)	0 (0%)
Canadian	8 (19%)	3 (0.9%)
Australian	1 (2%)	1 (0.3%)
Ghanaian	2 (5%)	0 (0%)
Nigerian	2 (5%)	0 (0%)
New Zealander	1 (2%)	0 (0%)
Scottish	1 (2%)	0 (0%)
Malaysian	1 (2%)	0 (0%)
Indian	1 (2%)	1 (0.3%)
Macedonian	1 (2%)	0 (0%)
Somalian	0 (0%)	5 (1.5%)
Pakistani	0 (0%)	5 (1.5%)
Filipino	0 (0%)	1 (0.3%)
Iranian	0 (0%)	7 (2.1%)
Malian	0 (0%)	1 (0.3%)
German	0 (0%)	2 (0.6%)
Eritrean	0 (0%)	2 (0.6%)
Djibouti	0 (0%)	1 (0.3%)
Bangladeshi	0 (0%)	3 (0.9%)

A study was conducted at the CPH to examine the effectiveness of incorporating cultural aspects into the curriculum by introducing a course-based cross-cultural interaction with a pharmacy school in Canada [32]. However, there was not enough information available about pharmacy educators' cultural competency in Qatar. Furthermore, the level of cultural awareness of pharmacy educators and the extent to which the curricular content was modeled for cultural compatibility in Qatar were unknown. The aim of this study, therefore, was to explore the cultural awareness of pharmacy educators and students at the CPH. The specific objectives were to (1) examine students’ perceptions about cultural diversity among educators and students, 2) identify students’ approaches toward cultural diversity, 3) determine whether students believed the curriculum appropriately emphasized cultural compatibility, and 4) examine the influence of students’ and instructors’ cultural diversities on pharmacy education in Qatar.

## Methods

### Study design and setting

A convergent, mixed-methods triangulation approach was used in this study which was conducted in the CPH at QU. Both quantitative and qualitative data were collected simultaneously to increase the validity and comprehensiveness of the study outcomes [33]. The Cultural Awareness Scale (CAS), a validated self-reported questionnaire, was used in the quantitative phase, while the qualitative phase utilized a constructivist, interpretative framework through four focus groups (FGs) conducted with undergraduate students. The results generated from the two phases were integrated and interpreted to reach an in-depth understanding of the topic.

### Quantitative phase

#### Population and sampling

The total population sampling approach, in which all pharmacy students in the BSc (Pharmacy) and PharmD degree programs were invited to participate in the study, was used in the quantitative phase. This method was applied because the target population of pharmacy students at the CPH was small (n = 122). The survey forms were emailed to all 122 students who were sent regular reminders to answer the questionnaire.

#### Data collection instrument

A thorough search of existing literature was conducted to find a suitable validated tool for the assessment of pharmacy students’ perceptions of cultural diversity among students and faculty members, and about the adaptation of curricular content for cultural compatibility in pharmacy education programs. Several tools such as the Cultural Awareness Scale (CAS) [34], Inventory for Assessing the Process of Cultural Competence Among Healthcare Professionals-Student Version (IAPCC-SV^©^) [35], Multicultural Assessment Questionnaire (MAQ) [36], California Brief Multicultural Competence Scale (CBMCS) [37], and Cultural Competence Assessment (CCA) [38] were identified. The IAPCC-SV^©^, MAQ, CBMCS, and CCA tools were excluded because they were focused on assessing students' perspectives about cultural awareness in clinical practice rather than in the teaching and learning environment. In addition, the CCA tools and the CBMCS were not tested in health professional education but were used in the general population of different organizations. The CAS was selected and adopted because it is the most comprehensive instrument that covers most aspects related to the objectives of this study, and it assesses students' perspectives about cultural diversity and awareness in the learning environment (i.e., the classroom and clinical practice). The CAS was designed and validated by Rew et al. (2003) to be used for measuring the multidimensional nature of cultural awareness in nursing students [34], and it was used in earlier studies to measure cultural awareness and cultural competence among both nursing [39, 40] and medical students [41]. The content validity of CAS was determined by a panel of experts in nursing and culture to yield a content validity index of 0.88 and a reduction of the total number of scale items to 36. To support construct validity, the scale was administered to 118 nursing students for factor analysis, and the combined data of the two samples revealed a Cronbach's alpha of 0.82 [34]. The final version of the CAS contained 36 items rated using a 7-point Likert-type rating scale (1 = strongly disagree to 7 = strongly agree). It measured cultural awareness in five different domains (general educational experience, cognitive awareness, research issues, behaviors/comfort with interactions, and patient care/clinical issues). The general educational experience (GEE) domain assessed cultural awareness and cultural competency application in education. The cognitive awareness (CA) domain assessed the effect of cultural diversity on students’ behaviors, attitudes, and beliefs. Furthermore, the research issues (RI) domain evaluated how well the college incorporates cultural factors in its research projects. The behavior/comfort with interactions (BCI) domain assessed the students' comfort and behavior while dealing with people from different cultural groups. Finally, the patient care/clinical issues (PCCI) domain assessed the cultural competency application and how students deal with cultural diversity in their own practice. The scoring system for each domain has been explained in Table 2. The total CAS score was calculated (CAS = 36 items; minimum = 36, maximum = 252). A higher score indicated a higher level of cultural awareness and cultural competence in education, while a lower score indicated less cultural awareness and competence [34]. In the absence of a standardized reference point in the original scale for high, moderate, or low cultural awareness, a method of consensus was applied by the current research team to consider a total CAS score range of 252–169 as high, 168–85 as moderate, and 84–0 as low.

**Table 2:** Cultural Awareness Scale (CAS) domains scoring system.

Domain	Number of items	Score (minimum–maximum)
General Educational Experience	14	14–98
Cognitive Awareness	7	7–49
Research Issues	4	4–28
Behaviors/Comfort with Interactions	6	6–42
Patient Care/Clinical Issues	5	5–35
Total CAS score	36	36–252

#### Data collection procedure

All 122 BSc (Pharm) and PharmD students at the CPH were invited to participate in the study via an email with a website link to the survey through the SurveyMonkey^®^ website. The survey was conducted from January, 2019 to March, 2019. A weekly reminder e-mail was sent to the target population over the two-month period to increase the response rate. The survey ensured complete anonymity of participants from whom no identifying information was collected. Participants were assured of confidentiality and data security measures.

#### Data analysis

The quantitative data obtained through the survey were analyzed using SPSS^®^ software (IBM SPSS^®^ Statistics for Windows, version 24.0; IBM Corp, Armonk, New York, USA) and SurveyMonkey^®^. Both descriptive and inferential analyses were performed appropriately. Descriptive statistics, including frequencies (%) and mean ± standard deviation (SD), were used to summarize the responses related to demographics and cultural awareness.

### Qualitative phase

#### Population and sampling

A purposive sampling approach was used, where students of diverse nationalities were selected from each batch to better achieve the study objectives. All the selected students were sent an invitation email to participate in the FGs. The email contained information about the objectives of the study and a copy of the Participant Information Sheet (PIS) that provided details about the research and the data collection procedures being used as well as a copy of the consent form that ensured confidentiality. The email was sent at least one week before the FG interviews were conducted for the selected students from each batch, and participation was confirmed by email. Because of the variation in students’ experiences, four homogeneous pharmacy student FGs were formed based on academic status—first professional year (P1), second professional year (P2), third professional year (P3), and fourth professional year (P4). PharmD students were not included in this phase owing to their limited availability in college due to clinical rotations outside the university.

#### Data collection instrument

A topic guide containing eight open-ended questions with probes for each question was used. The topic guide was prepared and conceptualized based on the research objectives and literature review [42].

#### Data collection procedure

Four face-to-face FGs were conducted with undergraduate pharmacy students for further exploration of their perceptions of the effect of cultural diversity among students themselves and between students and faculty members on the learning environment, process, and outcome. The FGs also explored how students dealt with this diversity, their perceptions of cultural compatibility in the pharmacy curriculum, and teaching and learning strategies in place. The FGs were conducted at the university by the researchers, while considering avoidance of bias and conflict of interest with participants, and ensuring consistency in data collection measures and approaches. At the beginning of each FG session, participants were informed about their rights as research subjects and were provided an informed consent form to read, understand, and sign. The signed forms were collected prior to the commencement of each FG by a FG facilitator. The FGs lasted between 45 and 60 minutes, and they were all conducted in English. All FG sessions were recorded using a digital audio recorder to allow the facilitators to focus on discussions while capturing all session details. Recordings of the FGs were transcribed verbatim by the researchers and were double-checked for accuracy. This transcription style ensured that the transcript was edited to filter out grammatical errors, distractions, pauses, incomplete words and sentences, or even fillers. Thus, an error-free, print-ready, readable transcript was ready, which was easily used for coding, analysis, and data extraction. To ensure preservation of confidentiality of data, transcripts were stored in a password protected laptop. Anonymization of all information in the transcripts was performed by using alphanumerical codes, with “P1”, “P2”, “P3”, and “P4” assigned to first, second, third, and fourth professional year pharmacy students, respectively. Credibility was maintained by sending a final version of the transcript to participants to ensure accurate reflection of their views.

#### Data analysis

Thematic analysis was performed by two independent researchers using NVIVO^®^ software. Both deductive and inductive thematic approaches were applied to organize the analytical priorities and examine data of indirect relations to the research question, respectively. Thematic analysis was performed by generating codes that were categorized into themes and subthemes. In cases of disagreements between data analysts, consensus was achieved through discussion.

### Ethical approval

Ethical approval for the study was obtained from the Qatar University Institutional Review Board (approval number QU-IRB 1014-EA/19). Participation in both phases was voluntary, and the respondents were informed that all the information they gave would remain confidential and anonymous.

### Informed consent

Written informed consent was obtained from all participants who participated in the quantitative and qualitative phases.

### Quality assurance

Mixed-method quality was evaluated by independently assessing the quality of each method [43].

#### Quantitative quality measures

The quality of the quantitative phase was assessed by ensuring the internal and external validity of the CAS tool. The CAS was psychometrically tested and validated, and, as mentioned previously, it has been used in previous studies to measure cultural awareness and cultural competence among nursing and medical students. In this study, only nursing-related words in the tool were replaced by those pertaining to pharmacy, thus ensuring that the tool’s validity remained unaffected.

#### Qualitative quality measures

The quality measures applied in the qualitative phase are provided in Table 3.

**Table 3:** Qualitative quality measures.

Measure	Application
**Credibility**	
Peer review	All steps were supervised by the research supervisors
Multiple source of data	Data were collected from four different FGs where each FG represents a different professional year (P1, P2, P3, P4)
Multiple participant categories	Data were collected from students from four professional years (P1, P2, P3, P4)
Proper data analysis strategies	Proper interpretation of data was ensured by conducting inductive and deductive thematic analysis
Member checking	Transcripts were sent to participants for verification
**Dependability**	
Full description	Full and detailed description of the methodology was written out
Database	A whole and comprehensive research database was available
Data triangulation	Data were collected from four different FGs were two researchers were involved (each researcher conducted 2 FGs)
Peer review	All research steps were audited by peer researchers
Inter-coder reliability testing	Data analysis for each group was performed independently and then compared between the researchers
**Conformability**	
Full description of methodology	Structured and detailed methodology were described and written to be followed
Database	A whole and comprehensive research database was available
Research process oversight	All steps were supervised by the research supervisors
Multiple sets of evidence	Data were collected from four different FGs
**Transferability**	
Recording the research details	Structured and detailed description about the FGs’ settings, contexts, and methods were provided
Data interpretation and discussion presentation	Results were described and interpreted in detail in the discussion
Member checking	Transcripts were sent to the participants
Integration between quantitative and qualitative data	Described in discussion section

P1: first professional year; P2: second professional year; P3: third professional year; P4: fourth professional year; FG: focus groups.

## Results

### Quantitative phase

Seventy students (57.9% of the target population) responded to the web-based survey. The distribution of students from P1, P2, P3, P4, and PharmD who participated in the survey is presented in Table 4. Students of different nationalities participated in the study, with a higher proportion of students from Egypt and Sudan (Table 4).

**Table 4:** Characteristics of surveyed pharmacy students at Qatar University.

Variable	N (%)
**Professional year**	
Professional year 1	18 (25.7)
Professional year 2	18 (25.7)
Professional year 3	14 (20.0)
Professional year 4	15 (21.4)
Pharm D	5 (7.1)
**Nationality**	
Qatar	5 (7.1)
Other gulf countries	1 (1.4)
Egypt	20 (28.6)
Palestine	7 (10)
Jordan	7 (10)
Syria	6 (8.6)
Sudan	12 (17.1)
Morocco	1 (1.4)
Algeria	3 (4.3)
Tunisia	1 (1.4)
Iran	2 (2.9)
Iraq	1 (1.4)
Others	4 (5.7)

The total CAS score represents cultural awareness and cultural competence in pharmacy education. The overall mean total CAS score for the study population was relatively high (187), with a maximum score of 251 and a minimum of 52. This indicated a relatively high level of cultural awareness among students. Individual item responses representing each domain of the CAS tool are presented in Table 5.

**Table 5:** Descriptive analysis of Cultural Awareness Scale (CAS) survey.

Item	Strongly agree	Agree	Somewhat agree	Neither agree nor disagree	Somewhat disagree	Disagree	Strongly disagree
Domain 1: General educational experience							
1-The instructors at this college of pharmacy (CPH) adequately address multicultural issues in pharmacy.	14 (20%)	28 (40%)	17 (24.3%)	7 (10%)	1 (1.4%)	2 (2.9%)	1 (1.4%)
2-This CPH provides opportunities for activities related to multicultural affairs.	12 (17.1%)	39 (55.7%)	13 (18.6%)	2 (2.9%)	3 (4.3%)	0 (0.0%)	1 (1.4%)
3-Since entering this CPH, my understanding of multicultural issues has increased.	12 (17.1%)	34 (48.6%)	12 (17.1%)	5 (7.1%)	3 (4.3%)	4 (5.7%)	0 (0.0%)
4-My experiences at this CPH have helped me become knowledgeable about the health problems associated with various racial and cultural groups.	17 (24.3%)	30 (42.9%)	17 (24.3%)	4 (5.7%)	1 (1.4%)	1 (1.4%)	0 (0.0%)
5-During group discussions or exercises, I have noticed the CPH instructors make efforts to ensure no student is excluded.	22 (31.4%)	28 (40.0%)	10 (14.3%)	6 (8.6%)	2 (2.9%)	2 (2.9%)	0 (0.0%)
*6-In my CPH classes, my instructors have engaged in behaviors that may have made students from certain cultural backgrounds feel excluded.	2 (2.9%)	4 (5.7%)	8 (11.4%)	8 (11.4%)	7 (10.0%)	23 (32.9%)	18 (25.7%)
^$^7-My instructors at this CPH seem comfortable discussing cultural issues in the classroom.	9 (13.0%)	23 (33.3%)	8 (11.6%)	8 (11.6%)	13 (18.8%)	5 (7.3%)	3 (4.4%)
8-My pharmacy instructors seem interested in learning how their classroom behaviors may discourage students from certain cultural or ethnic groups.	2 (2.9%)	17 (24.3%)	12 (17.1%)	25 (35.7%)	8 (11.4%)	3 (4.3%)	3 (4.3%)
9-I believe the classroom experiences at this CPH help students become more comfortable interacting with people from different cultures.	19 (27.1%)	36 (51.4%)	7 (10.0%)	6 (8.6%)	0 (0.0%)	0 (0.0%)	2 (2.9%)
*10-I believe some aspects of the classroom environment at this CPH may alienate students from some cultural backgrounds.	1 (1.4%)	10 (14.3%)	12 (17.1%)	20 (28.6%)	5 (7.1%)	18 (25.7%)	4 (5.7%)
11-My clinical courses at this CPH have helped me become more comfortable interacting with people from different cultures.	14 (20.0%)	35 (50.0%)	11 (15.7%)	5 (7.1%)	2 (2.9%)	2 (2.9%)	1 (1.4%)
12-I feel that the instructors at this CPH respect differences in individuals from diverse cultural backgrounds.	18 (25.7%)	41 (58.6%)	7 (10.0%)	2 (2.9%)	0 (0.0%)	2 (2.9%)	0 (0.0%)
13-The instructors at this CPH model behaviors that are sensitive to multicultural issues.	7 (10.0%)	13 (18.6%)	13 (18.6%)	25 (35.7%)	6 (8.6%)	5 (7.1%)	1 (1.4%)
14-The instructors at this CPH use examples and/or case studies that incorporate information from various cultural and ethnic groups.	13 (18.6%)	29 (41.4%)	14 (20.0%)	7 (10.0%)	6 (8.6%)	1 (1.4%)	0 (0.0%)
Domain 2: Cognitive awareness							
15-I think my beliefs and attitudes are influenced by my culture.	19 (27.1%)	23 (32.9%)	19 (27.1%)	2 (2.9%)	4 (5.71%)	1 (1.4%)	2 (2.9%)
16-I think my behaviors are influenced by my culture.	16 (22.9%)	23 (32.9%)	21 (30.0%)	5 (7.1%)	3 (4.3%)	0 (0.0%)	2 (2.9%)
17-I often reflect on how culture affects beliefs, attitudes, and behaviors.	11 (15.7%)	34 (48.6%)	13 (18.6%)	9 (12.9%)	2 (2.9%)	1 (1.4%)	0 (0.0%)
18-I believe pharmacist’s own cultural beliefs influence their pharmaceutical care decisions.	9 (12.9%)	21 (30.0%)	18 (25.7%)	6 (8.6%)	4 (5.7%)	10 (14.3%)	2 (2.9%)
19-I think students’ cultural values influence their classroom behaviors (e.g., asking questions, participating in groups, offering comments).	6 (8.6%)	16 (22.9%)	14 (20.0%)	10 (14.3%)	6 (8.6%)	14 (20.0%)	4 (5.7%)
20-I think it is the CPH instructor’s responsibility to accommodate students’ diverse learning needs.	21 (30.0%)	32 (45.7%)	9 (12.9%)	6 (8.6%)	0 (0.0%)	2 (2.9%)	0 (0.0%)
21-I think the cultural values of the CPH instructors influence their behaviors in the clinical setting.	3 (4.3%)	21 (30.0%)	18 (25.7%)	12 (17.1%)	9 (12.9%)	7 (10.0%)	0 (0.0%)
Domain 3: Research issues							
22-The faculty at this CPH conducts research that considers multicultural aspects of health-related issues.	8 (11.4%)	21 (30.0%)	10 (14.3%)	26 (37.1%)	2 (2.9%)	3 (4.3%)	0 (0.0%)
23-The students at this CPH have completed theses and dissertation studies that considered cultural differences related to health issues.	8 (11.4%)	12 (17.1%)	12 (17.1%)	29 (41.4%)	6 (8.6%)	2 (2.9%)	1 (1.4%)
24-The researchers at this CPH consider relevance of data collection measures for the cultural groups they are studying.	7 (10.0%)	11 (15.7%)	11 (15.7%)	36 (51.4%)	4 (5.7%)	1 (1.43%)	0 (0.0%)
25-The researchers at this CPH consider cultural issues when interpreting findings in their studies.	6 (8.6%)	13 (18.6%)	10 (14.3%)	35 (50%)	4 (5.7%)	2 (2.9%)	0 (0.0%)
Domain 4: Behaviors/comfort with interactions							
*26-When I have an opportunity to help someone, I offer assistance less frequently to individuals of certain cultural backgrounds.	1 (1.4%)	0 (0.0%)	3 (4.3%)	4 (5.7%)	5 (7.1%)	23 (32.9%)	34 (48.6%)
*27-I am less patient with individuals of certain cultural backgrounds.	0 (0.0%)	1 (1.4%)	6 (8.6%)	2 (2.9%)	6 (8.6%)	20 (28.6%)	35 (50.0%)
^$^28-I feel comfortable working with patients of all ethnic groups.	15 (21.7%)	33 (47.8%)	15 (21.7%)	1 (1.4%)	4 (5.8%)	0 (0.0%)	1 (1.45%)
*29-I typically feel somewhat uncomfortable when I am in the company of people from cultural or ethnic backgrounds different from my own.	1 (1.4%)	2 (2.9%)	7 (10.0%)	8 (11.4%)	12 (17.1%)	22 (31.4%)	18 (25.7%)
*30-I feel somewhat uncomfortable working with the families of patients from cultural backgrounds different than my own.	3 (4.3%)	4 (5.7%)	2 (2.9%)	4 (5.7%)	14 (20.0%)	28 (40.0%)	15 (21.4%)
31-I have noticed that the instructors at this CPH call on students from minority cultural groups when issues related to their group come up in class.	0 (0.0%)	5 (7.1%)	5 (7.1%)	29 (41.4%)	2 (2.9%)	14 (20.0%)	15 (21.4%)
Domain 5: Patient care/clinical practice							
32-I feel comfortable discussing cultural issues in the classroom.	6 (8.6%)	24 (34.3%)	19 (27.1%)	4 (5.7%)	10 (14.3%)	5 (7.1%)	2 (2.9%)
^$^33-I respect the decisions of my patients when they are influenced by their culture, even if I disagree.	23 (33.3%)	31 (44.9%)	9 (13.0%)	3 (4.3%)	3 (4.3%)	0 (0.0%)	0 (0.0%)
^$^34-If I need more information about a patient’s culture, I would use resources available onsite (e.g., books, videotapes).	14 (20.3%)	26 (37.7%)	19 (27.5%)	4 (5.8%)	4 (5.8%)	2 (2.9%)	0 (0.0%)
35-If I need more information about a patient’s culture, I would feel comfortable asking people I work with.	14 (20.0%)	35 (50.0%)	14 (20.0%)	3 (4.3%)	2 (2.9%)	0 (0.0%)	2 (2.9%)
36-If I need more information about a patient’s culture, I would feel comfortable asking the patient or family member.	16 (22.9%)	35 (50.0%)	13 (18.6%)	2 (2.9%)	3 (4.3%)	1 (1.4%)	0 (0.0%)

* Items with reversed scores.

$ Items with a missing response.

Abbreviation: CPH, college of pharmacy.

For the GEE domain, all groups had relatively high mean scores. The overall mean score for this domain was 73 (maximum score = 98, minimum score = 19). Descriptive data showed a high rate of agreement and a low disagreement rate for the items under this domain. These results indicated that there was an adequate application of cultural awareness and competency in pharmacy classes, and that the students had a culturally sensitive education experience in the CPH.

For the CA domain, different student groups had very close mean scores with the highest score in the PharmD group. The overall mean score for this domain was 36.3 (maximum score = 49, minimum score = 10). Similarly, descriptive data showed high rates of agreement for the items under this domain. Individual item responses related to this domain showed that the students believed that their behaviors, attitudes, and beliefs are generally influenced by their cultural backgrounds.

For RI, the overall mean score was 18.9 (maximum score = 28, minimum score = 7). Descriptive data showed differing results for this domain; agreement rates were lower than those for other domains. Agreement rates for this domain were between 10% and 30%, and there were higher percentages of students who chose to be “neutral.” The results revealed that factors pertaining to cultural diversity might not have been well incorporated in research projects and research questions, a shortcoming that needs to be considered in future research projects in the college.

For the BCI domain, the overall mean score was 31.84 (maximum score = 41, minimum score = 7). Most of the items in this domain have reversed scores, where a lower percentage rate represents a higher level of awareness. The descriptive statistical data showed high rates of disagreement for the reversed items and high rates of agreement for the normal ones. The findings indicated that the surveyed students believed they were able to deal with people from different cultural groups and did so comfortably.

For the PCCI domain, the overall mean score was 27.9 (maximum score = 35, minimum score = 9). The descriptive data analysis showed high rates of agreement for the items of this domain. These results indicated that the students believed that they were culturally aware and sensitive while dealing with patients from different cultural backgrounds during their experiential training and practice.

### Qualitative phase

Details about the number of students who participated in each FG, are provided in Table 6. All participants were 19–25 years old Muslim females. P1 FG included 1 Tunisian, 2 Pakistanis, 1 Syrian, and 1 Sudanese students. While P2 FG comprised 1 Qatari, 2 Sudanese, and 3 Egyptians. P3 FG participants were from Syria (1), Sudan (2), Palestine (1), and Egypt (2). P4 FG participants were from Qatar (1), Palestine (2), Iran (2), and Morocco (1). Several themes and sub-themes emerged from the FGs; five of the themes were deductive, while two were inductive. The themes and sub-themes generated are shown in Table 7.

**Table 6:** Number of students invited/accepted to participate in FGs.

	Number of students invited	Number of students who participated
P1	11	5
P2	12	6
P3	12	6
P4	11	6

P1: first professional year; P2: second professional year; P3: third professional year; P4: fourth professional year.

**Table 7:** Themes and subthemes generated from the FGs.

Theme approach	Themes	Subthemes
**Deductive Themes**	Cultural diversity among pharmacy students, and among students and faculty	Academic performance
		Communication and relationships
		Benefits and opportunities
	Faculty cultural awareness and its demonstration in teaching	Practical application
		Barriers of application
		Language
	Dealing with unawareness	
	Curriculum modifications to address cultural diversity	Cultural competency aspects
		Introducing Arabic language
	Effect of cultural diversity on Qatar’s values and heritage	Balancing development and traditions
		Increasing English language use
**Inductive Themes**	Religion and science	
	Society’s reactions	

#### Deductive themes

*Cultural diversity among pharmacy students and between students and instructors*. This theme addresses the effect of cultural diversity on students and faculty. Three sub-themes emerged:

*Academic performance*. Most participants agreed that the cultural diversity among students themselves and between students and faculty did not affect the academic performance of the students. One participant said:

"Almost all of us [have] studied in the same schools and we are [taught] the same academic subjects here in the College of Pharmacy. So, it does not affect our academic performance. So I would say cultural diversity is a positive thing." P3-2

One student explained that academic performance depends on the student herself and the effort she puts in for better academic performance, and that cultural diversity has no association with the students' academic performance. She said:

"Academic-wise, it's more likely [to be] related to personal issues, like your effort while studying. Culture does not have a relation with that. You can see two girls from the same culture, but with different academic performances." P3-5

*Communication and relationships*. Miscommunication between students and instructors may occur because of cultural diversity and differences in their native languages and dialects. A student whose native language was Persian stated:

"Sometimes, the communication between instructors and I was hindered because of our differences [in] languages and cultural backgrounds. For example, when instructors asked me a question, I knew the answers in Persian, but I did not know how to express my ideas and thoughts in English. This makes the interaction difficult." P4-4

Another student said:

"If we are talking to an Arabic instructor, it's easier to understand each other not only because of the [common] language but [also] because we have a common culture, while it is harder to talk to instructors from western nationalities and explain to them what you want." P3-2

*Benefits and opportunities*. There was agreement among students that cultural diversity creates an opportunity for them to learn about different cultures, traditions, and backgrounds. One participant explained:

"I think when I sit with my friends from different cultures and ask them about their cultures, I would learn from them. It is a good opportunity to learn from other students about their cultures. I feel I [have] gained great knowledge from them." P4-6

Additionally, instructors from Western countries with different cultures provided students with opportunities to learn about different practices and beliefs that will prepare them to work in a multicultural society in the future. A student said:

"The communication with people from different nationalities and religions will prepare us for[our] future careers. For example, [it will improve our] communication with patients in practice. P1-3

Another student agreed with what was said, adding:

"You need to be exposed to different perspectives and different kinds of people, and you need to be more open to other cultures to be able to deliver the kind of care you are trained to give. I think that having professors from different cultures facilitates this concept because when you go to practice you will deal with different cultures.” P4-1

*Faculty’s cultural awareness and its demonstration in teaching*. This theme explores the cultural awareness of the faculty members in the CPH and their application of awareness while teaching students. Three sub-themes emerged:

*Practical application*. Recognition of general local cultural identity and values, as presented earlier, by instructors was illustrated in students’ views. A student explained:

"I guess all the instructors, even the western ones, know about our cultural values and are well adapted to it. For example, whenever they are talking to us, they know that we do not shake hands. Whenever we are fasting, they accept Ramadan regulations. They also excuse our missing from classes because of the prayers.” P3-3

On the other hand, a student mentioned that some instructors were theoretically aware of Qatar’s culture, but they did not reflect that awareness in their actions. She stated:

"I would say that instructors are aware of cultural diversity, but they don’t always act on it. They teach about cultural diversity and how to be culturally sensitive, but they [themselves] are not or they may not be very culturally sensitive to the major[ity] culture here in Qatar.” P4-2

With regard to demonstrating awareness in teaching, a student said:

"There was a session on professional skills about cultural competency. I remember that we had different activities where we had standardized patients who believed in traditional medicines. We were taught to keep cultural diversity in mind while interacting with them and to respect their beliefs." P3-1

*Barriers to application*. Notably, instructors’ application of cultural awareness was impeded by some barriers, as described by students. One such issue was instructors’ hesitancy to communicate and interact with students on culturally sensitive topics. For example, one student explained:

Some faculty members are very eager to know about our culture, but at the same time they are very afraid to ask questions and open up with the students. For example, when some students try to discuss some sensitive issues, some instructors try to escape from the concept because they do not want to get in trouble." P4-4

Another barrier was that instructors coming from different cultures and backgrounds assumed that all Arabic and Muslim students have the same culture, beliefs, and traditions. One student said:

"The instructors have lived their whole lives in their own countries, and they have one image about Arabs and Muslims. So, we all have been categorized in the same category from their perspective. They assume that all students have similar cultures, values, and backgrounds and treat all students similarly based on these assumptions." P4-1

Some participants stated that the university played a role in the instructors' reluctance toward discussing cultural issues with the students and opening up with them. They stated:

“The culture[al] differences [are] not the major problem that hinders the instructors’ openness or engagement with the students, but I think whoever explained the gender and other cultural barriers to them had possibly took it to another level that the instructors felt that they could not connect with us because of these differences." P4-1

Another student stated:

“Another message that was conveyed to the instructors is that we are too sensitive [about] culturally sensitive topics. So that makes them more hesitant about communicating these issues with us. So, [they are] not to blame.” P4-2

*Language*. This sub-theme highlighted practices adopted by instructors regarding language differences. While some students pointed out that instructors usually took into consideration language differences during teaching, others showed how information could be unequally transferred to students because of differences in languages used to communicate with each other, thus, affecting their learning. For instance, a student said:

"Faculty take into consideration language differences and ask if there is anyone who cannot understand Arabic if they want to explain or mention something in Arabic. They stick to English language when they know that there are non-Arabic speakers in the class.” P4-5

On the other hand, some students complained that some instructors did not take language differences into consideration. A non-Arabic speaking student said:

"Some professors prefer to speak in Arabic even when we tell them that we don’t understand them. Sometimes, they just start speaking Arabic by default. It would be better if they ask if there is someone who does not understand Arabic." P1-2

Another non-Arabic speaking student added:

"Sometimes, when the students ask questions in Arabic, the instructors explain the answers in Arabic based on the question language, and they don't translate it to English. However, I think it is our right to have these questions and answers translated to [English to] get the benefit the other students [receive]. " P1-3

Another student commented on having only a minority of class students as non-Arabic speakers:

"I think our class to some extent is diverse but having only two non-Arabs in the class is a barrier, because sometimes the instructors start speaking [in] Arabic without making sure that everyone is understanding. Non-Arabs will feel left out." P1-5

*Dealing with unawareness*. This theme discusses some situations of cultural unawareness that students experienced and their reactions to these situations. A student described one situation as follows:

"In the white coat ceremony, I was surprised that a male doctor will [make me] wear the [ceremonial] coat, which is not acceptable for me and my family. Most of the students complained to the college about this." P3-5

Another situation was discussed by one of the participants:

"Last year we were discussing some ethical points of view. It was about end-of-life care. The professor was not fully aware of our culture, but he allowed us to debate [with] him and we had a very productive conversation." P3-1

A participant said:

"One of the IPE events was discussing mental health and how our culture relates some mental diseases to religion. This discussion was stopped early since some of the instructors and students were not Muslims and they do not believe in this theory. I believe that there are diseases that require medical treatment as well as spiritual or religious healing, which I do not think they are aware of. I actually ignored what happened and I did not try to clarify what I believe personally." P4-2

Another student stated:

“Sometimes, I feel some students speak in Arabic in classes without noticing that the non-Arab instructors sometimes get bothered since they do not understand if there is a question or problem. I think this is unawareness from the students to[ward] the instructors. When this happens, we ask them to speak in English because it is more respectful to the instructors.” P2-1

With regard to dealing with unawareness issues, one student described:

"It depends on the situation. If it was a major thing, I would discuss it, but if it was a minor thing I would ignore it and with time they will learn about it." P3-2

*Curriculum modifications to address cultural diversity*. This theme explores students' suggestions for curricular modifications and improvements to make the curriculum more culturally sensitive and compatible. Two subthemes emerged:

*Cultural competency*. The students agreed that more material related to cultural competency and dealing with people from different cultures needed to be added to the curriculum. A student stated:

"We had a class last year about cultural competency in professional skills; however, this is not enough. We need to be exposed to more cultural issues since we will experience more of these issues in SPEP [Structured Practical Experiential Program] rotations." P3-4

One student commented:

"I think we need to move from cultural diversity as a subject or a lecture to a real application. For example, in the professional skills lab, incorporating Arabic language and even some words from other languages, like Indian, to be able to deal with the huge number of patients who do not speak Arabic or English, and to know how to deal with them [is needed]." P2-4

Another student suggested:

"It will be beneficial if there is research assessing what major cultural differences exist in Qatar’s health care system and community, and then we get these cultural issues included in the curriculum." P3-5

With regard to the standardized patients (SPs) in professional skills, a student explained:

"We always had been exposed to SPs who are educated and able to understand what you are saying and can repeat your recommendations, but it was totally different in SPEPs; we had patients who don’t easily understand, patients who refuse the recommendations and negotiate them, and even [those] who cannot speak English or Arabic. We would like to see such cases in the professional skills sessions. It would be beneficial to get to the sense of how to deal with these cases." P3-4

*Introducing Arabic language*. The importance of introducing Arabic language in some parts of the curriculum was clearly evident in students’ discourses. Some participants mentioned:

"I think it would be beneficial to add some Arabic translation in the lectures or at least add the diseases names. Also, adding Arabic stations in professional skills sessions would be beneficial." P4-2

"We don’t even know the Arabic names of the medical conditions we learn [about]. Therefore, if my family asked me about any condition, I would not even know what it is [called in Arabic]. So, I think they need to involve Arabic vocabularies to make communication easier in the practice." P2-2

Many students described the perceived difficulty of interacting with Arabic-speaking patients because the curriculum was based on the English language with no supplementary materials in Arabic. Some participants stated:

"I remember I had to talk in Arabic in my first interaction with a patient in my SPEP. I stopped talking and stopped asking because I could not ask [questions] in a flowing way, which affected the care provided to him." P3-4

"I was in cardiology in one of the SPEPs and I wanted to counsel a patient about INR [International Normalized Ratio]. I could not explain it to the patient because I did not know the terminologies in Arabic." P4-4

One student suggested:

"They can give us more chances to express ourselves in Arabic, for example, in the debates, [or] journal clubs. If the professor speaks Arabic and all students understand Arabic language, I would like to give my opinion in Arabic. If the doctor understands Arabic, he/she can at least summarize the lecture in Arabic for the sake of understanding.” P3-1

*Effect of cultural diversity on Qatari values and heritage*. This theme demonstrates the effect of cultural diversity on Qatar's traditions, values, and heritage and it comprises two sub-themes:

*Balancing development and traditions*. The participants agreed that the burgeoning cultural diversity with the development in Qatar should not change its core values, cultural identity, and heritage. They stated:

"Qatar is getting more international as it is holding international events such as [the] 2022 World Cup. So, I see that there is more opening[s] for different cultures, but with no [significant] changes of [its] heritage and values.” P3-2

"People are trying to [strike] a balance between traditions and what is coming from outside. The society, 20 years ago, was stricter, but now you need to be open since it is overwhelmed with different people and cultures. We can be open to that, but we also have to stick to our beliefs that we were raised on." P3-3

Another participant commented on the transformation witnessed in public institutions from purely gender-segregated environments to uniquely conservative mixed gender settings:

"Before, there was segregation between the males and females here, in Qatar. However, now there is more blending between males and females in some fields and experiences, but still there is the issue of regulating our interactions." P3-3

*Increasing English language use*. Due to the linguistic diversity among students themselves and between the students and instructors, some participants stated that English is now being used more often, even by Arabic-speaking students outside the classroom. For example, one student mentioned:

"Unfortunately, [the] English language is being used even by Arabic students. For example, now I text with my friends and talk to them in English because we talk in English a lot in the classes, so we get used [to it]and do it outside." P3-1

Another student commented about the diversity of Arabic dialects:

“I feel more comfortable speaking in English than in Arabic, because I don’t understand some of the other [Arabic] dialects. That is why I also speak to Arabic instructors in English. Speaking in English is needed as it will make conversation easier because of the diversity of Arabic dialects." P3-3

A P1 student mentioned:

"Speaking in English in class or outside the class will not affect the Arabic heritage of this country, because English is a universal language everybody understands. I mean some English words are known by most of the people here to allow them to communicate with others." P1-5

#### Inductive themes

*Religion and science*. There was a perception among students that many instructors who came from Western countries, due to their limited understanding of the local culture and religion, think that Muslim students are not allowed to learn about some sensitive topics owing to religious restrictions. They stated:

“Religion and culture are two different aspects. Things could be religiously appropriate and culturally inappropriate. For example, we come from [the] Arab world and we are all Muslim and we follow Islam. But every country has its own culture even though we have one religion.” P4-3

“Our religion does not prohibit us from learning about stuff that we need in life. However, the instructors may think that our religion is too conservative and that you will not [want to] learn anything about sexual diseases, for example. In contrary, our religion has given many ways of learning [about such topics] and getting the education that we need.” P4-1

*Society’s reactions*. Students felt that they were judged as being culturally insensitive or too open-minded when they discussed or counseled patients about sensitive topics during their SPEPs. However, students justified these practices as being a result of a professional attitude. Students explained:

“As students here, we have learned how to be professional and when we are trying to approach patients and counsel them about sensitive topics some of them used to get shy not only because of their conditions, but also because we are proactive to discuss and counsel them without feeling shy about it. So, when I was in practice, it was very hard to convince female [patients] on consulting the pharmacist about the medication they need to take.” P4-3

“Another thing is that sometimes when you are so proactive talking about sensitive topics so openly, people judge your character. There are some situations that happened to me where some people judged me, thinking that I lack ethical standards because I am open to talk about these sensitive topics” P4-4

Another participant commented:

"I think we cannot go so deep while discussing specific topics, but I don’t know, maybe by mistake, you can express your opinion based on your culture, [and] at the same time you are offending the others who have different beliefs or different values. I think these kind[s] of subjects make differences where we seem that we do not respect the person or his/her culture." P2-2

## Discussion

This research is the first step in exploring the level of cultural awareness among instructors and students at the Qatar University CPH from the students’ perspective. It sheds light on the influence of students’ and instructors’ cultural diversity on Qatar’s pharmacy education. Although the CPH can be considered a small multicultural community in Qatar, it is worth mentioning that the demographic structure of Qatar and that of the CPH’s population differs significantly; while the majority of Qatar’s population comprises expatriates from India and Bangladesh, the CPH community is made up largely of Arab students (89%) and non-Arab western instructors (67.5%). While it can be argued that the CPH’s student body is a homogenous Arab female cluster, this study found deep cultural diversity among people with similar Arabic identities.

The quantitative and qualitative data were compared and it was found that the quantitative data demonstrated an overall high level of cultural awareness and competency in pharmacy education at the CPH. However, the qualitative data disclosed the areas of suboptimal cultural awareness. In fact, the students’ cultural awareness is high compared to the perceived level of cultural awareness among nursing students in Sweden [44]. This is consistent with Rew’s (2003) study, which said that educational environments with high cultural diversity reflect a high degree of cultural awareness and sensitivity in their students and instructors [34].

The present study adds to the existing body of evidence that culture influences individuals’ beliefs, behaviors, and attitudes [45]. This is important, as pharmacy graduates will undoubtedly interact with healthcare providers and patients from different cultures who possess beliefs, values, and attitudes about health and illness that are different from theirs. As disparities in healthcare may result from inappropriately addressed cultural differences between patients and pharmacists [46, 47], minimizing these disparities by educating students about the influence of sociocultural norms on health and beliefs, and equipping them with skills to become culturally competent, are widely suggested [2, 48]. Interestingly, CPH students perceived their recognition of cultural diversity, and their ability to interact with people of different cultural backgrounds, as correlative with having a high level of confidence. This can be explained by the benefits they received from the opportunities provided to them in a culturally diverse educational environment. Notably, a variation in perceived ability to deal with cultural diversity was observed among students in the lower and upper professional years. This could be due to the differences in levels of experience between the two groups, since the upper professional year students were more exposed to life situations than the lower professional year students. However, students’ discussions reflected they lacked awareness and competence in some areas, such as recognizing the major cultural differences existing in various communities in Qatar and their foreign languages and dialects. This is congruent with the study by Murden et al., who argued that most of the occupational therapy students perceived their knowledge about different cultures to be limited [49]. Moreover, taking into consideration that the majority of students are Arabs, the study’s qualitative evidence showed some intentional and unintentional instances of Arabic communication in the presence of non-Arabic professors or students, which was perceived negatively by the latter. In addition, students’ preference to communicate and interact with Arab instructors over non-Arab ones was also evident.

The current study also demonstrated the students’ self-perception of high appreciation and consideration of patients’ diverse cultures and backgrounds during experiential training. This aligned with Cooper’s (2014) study, which was conducted on P4 pharmacy students at Midwestern University Chicago to assess the students’ perceptions of cross-cultural experiences during experiential training as well as the perceived comfort levels with various cultural encounters [50]. More than half of the surveyed students declared their comfort while providing culturally competent care to patients with diverse beliefs and behaviors, especially those afflicted with disabilities, financial problems, and mental health diseases [50]. However, an accurate assessment of students’ comfort with providing culturally competent care could not be ensured by self-assessment only. To address this concern, it was suggested that the curricula be modified to adapt preceptors’ involvement in observing and providing feedback to students [50]. Furthermore, this study provides a means of societal involvement in assessing students’ cultural awareness, sensitivity, and competence. A disagreement was noted between societal and students’ perceptions of the students’ cultural awareness levels during culturally sensitive encounters or discussions. While pharmacy students believed that they behaved and interacted professionally, healthcare practitioners and patients judged them as being culturally insensitive or too open-minded. This controversial fact supported the recommendation in a previous study for incorporating a cultural sensitivity component to the newly introduced cross-cultural interaction course at the CPH [32]. On the other hand, a recent study that aimed at exploring how Australian pharmacists perceived culture and cultural awareness in their practice reported cases where they appreciated how pharmacy students were open to different cultures and thoughts [51]. The pharmacists also commended the highly desirable trait of self-awareness and cultural awareness exhibited by students, as they tremendously enriched the practice [51]. Therefore, an examination of patients’ and healthcare practitioners’ perceptions of the interaction and communication of pharmacy students in culturally sensitive cases ought to be further investigated [34].

This study also demonstrated that even though cultural awareness and competence among the CPH’s instructors were rated highly in the majority of CAS items, students disclosed some instances of instructors’ cultural unawareness and barriers that hindered instructors from demonstrating awareness while teaching. These include non-Arab instructors’ possession of generalizable assumptions about similar cultures. In a comparable qualitative study, pharmacy students at the University of Illinois voiced their concerns when faculty lumped different races and ethnicities together, and pointed out the fact that not all “African-American” students preferred to be addressed as “Black” when discussing the pharmacotherapeutic aspect of a certain disease [42]. The fact that around 30% (8 of 27) of the non-Arab CPH faculty members had been hired recently, between 2017 and 2019, might explain the observed poor cultural awareness among members of this group. This might not be a sufficiently long period for them to be exposed to Arabic culture and Islamic values to recognize the Islamic attitude towards sciences, or to confidently handle cases of a culturally sensitive nature as compared to instructors with longer experience.

Additionally, exploring the relationship between cultural diversity, instructors’ level of awareness, and students’ academic performance suggested that cultural diversity did not play a direct role in influencing academic performance; however, it was associated with miscommunication, which compromised general understanding and learning. This finding was supported by a recent qualitative study that showed that culture is considered to be one of the factors that influence pharmacy students’ levels of engagement, learning, and academic performance [52]. Also, as clearly observed, linguistic differences were perceived as one of the major barriers affecting the transfer or reception of knowledge, and, hence, the quality of the learning experience. The study’s qualitative phase indicated that there have been instances where instructors used Arabic in classroom activities that affected the learning of the minority non-Arab students and cases of students expressing an inability to interact with English-speaking instructors. Poor interactions between instructors and students as an overwhelming problem impacting the learning experience was also witnessed among culturally and linguistically diverse nursing students in another study. [53]. This study demonstrated that even if students had language skills that allowed them entry into college, these skills might not have been sufficient for some students to receive the guaranteed benefits of learning opportunities, assertive communication, and successful integration.

The current study participants reported proper implementation of cultural competency and expertise in the curriculum and courses of this Middle Eastern College of Pharmacy that offered students a simulated professional skills course series and real encounters with culturally diverse patient populations in SPEPs. The college curriculum reflects the US Accreditation Council for Pharmacy Education’s 2016 guideline, which states that pharmacy colleges and schools in the US shall consider cultural appreciation in their curricula and ensure that graduates are capable of incorporating cultural diversity in their practice [3]. The literature demonstrated a limited variety of approaches to teaching and assessment to implement cultural competence courses and training in pharmacy and other health profession educational programs [54–57]. This was evident in a recent collaborative study that was conducted between the CPH and the University of Saskatchewan, where the investigators developed, implemented, and evaluated a course-based cross-cultural student interaction [32]. This innovative method showed an effective way of inducting cultural aspects into the students’ process of acquiring knowledge, where they interacted with each other on case-based discussions, exchanging their experiences [32]. Despite these attempts, participants of this study stressed the importance of further curricular modifications to gain deeper insight into cultural competency. The assessment of the presence of cultural competency content in Canadian and American pharmacy school curricula has shown that most curriculum committee heads in the surveyed schools recognize the importance of incorporating content pertaining to cultural competency; however, only a few comprehensively made these curricular changes [58]. This shows that the cultural competence content in the international health professional educational curriculum is scarce.

Another point that demonstrates the insufficiency of cultural components in the CPH’s curriculum is the absence of, or insufficiency in, training students to provide linguistically appropriate care. The participants pointed out their shortcomings while interacting with Arabic-speaking patients and recommended that the curriculum incorporate Arabic terminology and descriptions. This suggestion can be contextualized by examining the CPH database records, which indicates that the majority of CPH graduates are currently employed in Qatar. A similar problem was observed in the College of Pharmacy at the University of Sharjah (UAE), where most of the pharmacy students enrolled were native Arabic speakers while the medium of teaching is English [59]. These students expressed their desire to receive proper training in Arabic communication, especially for using medical information in future encounters with Arabic-speaking patients. An initiative to address this issue was taken by introducing Arabic content in five, three-hour long tutorials that were added to the communication and clinical skills course. Assessment of the usefulness of this curricular change to adapt to the UAE’s culture revealed a positive impact on students who felt they had enhanced their communication skills [59]. Since Qatar and the UAE have similar cultural ethos, and the fact that almost one-third of the total population of Qatar comprises Arab nationals, the application of such a change in the CPH is expected to yield favorable learning outcomes and expertise in students. The relationship between health professional language competence and patients’ health outcomes was investigated revealing that the proper use and comprehension of language between healthcare providers and patients could effectively improve health outcomes for patients and reduce errors in treatment [51, 60].

Another interesting finding of this study was the partial demonstration of “cultural inclusiveness” in the CPH’s educational environment. This was elicited from the equal rates of agreement and disagreement in students’ responses to item 10 under the “general education experience domain” of the CAS, which indicated that there could be some instances of alienation from some cultural backgrounds in the classroom environment. This was also supported by students’ discourses wherein they perceived minority populations (i.e., non-Arabs) as a barrier to the process of teaching, which could result in occasions of unequal treatment toward minorities, hence compromising their sense of belonging. The perception of cultural inclusiveness was examined among medical students at James Cook University over a period of four years revealing similar findings, with a low rating of institutional intercultural inclusiveness [61] reported. The students perceived low satisfaction with respect to the efforts of their instructors to understand their diverse cultural needs and insufficient opportunities were provided to them to learn about cultural differences. The study highlighted the need for professional development interventions to improve cultural inclusiveness among faculty members [61].

Furthermore, participants indicated that cultural factors were not considered or adequately integrated in research designs and projects conducted at the CPH. This finding is congruent with that of Safipour (2017) and Rew (2014), who showed that nursing students demonstrated low cultural awareness related to research issues because of their limited experience [44, 62]. While culture has become more relevant in health studies as it influences physical, mental, and emotional wellbeing, and overall health choices [63], healthcare researchers are challenged with understanding cultural dimensions and how their variations can be considered in health research [64]. Al-Bannay (2014) pointed out that researchers in health disciplines are not trained as interculturists, which hinders them from considering cultural factors in their research and benefiting from cultural knowledge [64].

Finally, the findings emphasized the uniqueness of the multi-cultural society of Qatar and also the importance of maintaining Qatar's traditions along with a spirit of development. Although some scholars might debate that Qatar has introduced many initiatives and measures in the QNV 2030 to protect and preserve its cultural identity from multi-culturalism [27, 65], there are others who believe that the deepening cultural diversity that exists in educating and training healthcare professional students in Qatar might have impacted the cultural values and identities of students [24]. It was evident that the cultural diversity experienced in the pharmacy program at QU compromised the expression of some Qatari cultural features and attributes. One of these is the preferential treatment and the overuse of English over Arabic despite the huge reinforcement for the use of the Arabic language in governmental and non-governmental activities and events stipulated in Law No. 7/2019 by Amir HH Sheikh Tamim bin Hamad Al Thani. Many QU scholars perceive this law as a measure that supports Arabic and protects Qatar’s own culture [66]. In addition, there has been a new trend of integrating both genders in the QU’s health college events, such as in inter-professional education (IPE) events and in health-related conferences, symposiums, and forums. Another major example of new gender-integrated educational programs at QU is that of the College of Medicine. However, undergraduate education at QU, governmental education in Qatar, and the majority of public universities in the Gulf region are gender segregated [67, 68]. While gender integration in health education programs provides the opportunity for students to overcome the challenges of interacting with healthcare professionals and patients of the opposite sex in the future [68, 69], this is considered a cultural barrier for some conservative students, which might affect the quality of their learning experience in such nontraditional environments [69]. Therefore, this study highlights the need for educators, researchers, and policymakers to work together toward culturally sensitive approaches to health profession education based on Qatar’s Islamic and Arabic identity [70].

Although the study was conducted on a small sample size compared to other studies [62, 71], meaningful insights about cultural awareness among pharmacy students and educators were obtained. The mixed-methods approach allowed researchers to gain the advantages of the amalgamated quantitative and qualitative modalities, thus yielding a better understanding and a more holistic set of evidence [72]. The first modality used the CAS tool, which is a validated tool proven to accurately measure the perception of nursing students about cultural sensitivity [34, 62]. The second modality utilized a FG approach, which ensured participants’ openness and reflection on their personal experiences. Additionally, triangulation was achieved using several sources of data, which facilitated the production of valid and comprehensive findings [72]. On the other hand, this study has some potential limitations. This research was conducted in the pharmacy college with a small population, which restricts the generalizability of findings to other colleges. Moreover, the study cohort was statistically underpowered, which limited the cultural awareness comparison between pharmacy students in different professional years. In addition, the CAS was a self-administered questionnaire; therefore, a possibility of response bias and/or exaggeration of results or missing details might have occurred.

## Conclusions

The internationalization of pharmacy education in Qatar has introduced the students and the educators to new dimensions of awareness of cultural diversity. For pharmacy students and educators, cultural awareness and competence during classroom courses and experiential learning has been a mixed blessing. Although cultural diversity was considered a great learning opportunity to prepare students for future practice in their profession, some challenges and barriers hindered culturally sensitive communication and attitudes, thus affecting the quality of the learning experience at the CPH. While the college attempted to provide culturally sensitive educational opportunities to students, it proved insufficient to prepare them to be culturally competent healthcare practitioners.

As this research is the first attempt to examine the influence of Qatar’s cultural diversity on pharmacy education, suggestions for future studies include investigating the instructors’ self-perception of their knowledge, attitudes, and behaviors regarding cultural diversity and its impact on health professional education. This will facilitate the optimization of cultural competence-related educational interventions for both instructors and students. In this regard, local health profession educational programs are advised to reassess their curricula for the adequacy of theoretical and practical cultural competence content and the potential to maintain Qatar’s culture and identity, while ensuring cultural inclusiveness. It is also recommended that research be conducted to enhance understanding and application of the most effective teaching strategies of cultural competence in the health profession field. Importantly, exploring perspectives of culturally diverse patients in Qatar on healthcare provisions, and the extent of cultural awareness and sensitivity of healthcare practitioners in Qatar, is highly recommended to improve healthcare services.
